# Cost-effectiveness of cryotherapy versus salicylic acid for the treatment of plantar warts: economic evaluation alongside a randomised controlled trial (EVerT trial)

**DOI:** 10.1186/1757-1146-5-4

**Published:** 2012-02-27

**Authors:** Eugena Stamuli, Sarah Cockayne, Catherine Hewitt, Kate Hicks, Shalmini Jayakody, Arthur Ricky Kang'ombe, Gwen Turner, Kim Thomas, Mike Curran, Farina Hashmi, Caroline McIntosh, Nichola McLarnon, David J Torgerson, Ian Watt

**Affiliations:** 1 Department of Health Sciences, York Trials Unit, University of York, ARRC Building, York YO10 5DD, UK; 2 Centre of Evidence Based Dermatology, University of Nottingham, Nottingham, UK; 3 School of Health, University of Northampton, Northampton, UK; 4 4 University of Brighton, School of Health Professions, Brighton, UK; 5 The National University of Ireland, Galway, Discipline of Podiatry, Galway, Ireland; 6 Glasgow Caledonian University, School of Health and Social Care, Glasgow, UK; 7 Hull York Medical School, York, UK

**Keywords:** Plantar warts, Verrucae, Cost-effectiveness analysis, Salicylic acid, Cryotherapy using liquid nitrogen

## Abstract

**Abstract:**

**Background**

Plantar warts (verrucae) are extremely common. Although many will spontaneously disappear without treatment, treatment may be sought for a variety of reasons such as discomfort. There are a number of different treatments for cutaneous warts, with salicylic acid and cryotherapy using liquid nitrogen being two of the most common forms of treatment. To date, no full economic evaluation of either salicylic acid or cryotherapy has been conducted based on the use of primary data in a pragmatic setting. This paper describes the cost-effectiveness analysis which was conducted alongside a pragmatic multicentre, randomised trial evaluating the clinical effectiveness of cryotherapy versus 50% salicylic acid of the treatment of plantar warts.

**Methods**

A cost-effectiveness analysis was undertaken alongside a pragmatic multicentre, randomised controlled trial assessing the clinical effectiveness of 50% salicylic acid and cryotherapy using liquid nitrogen at 12 weeks after randomisation of patients. Cost-effectiveness outcomes were expressed as the additional cost required to completely cure the plantar warts of one additional patient. A NHS perspective was taken for the analysis.

**Results**

Cryotherapy costs on average £101.17 (bias corrected and accelerated (BCA) 95% CI: 85.09-117.26) more per participant over the 12 week time-frame, while there is no additional benefit, in terms of proportion of patients healed compared with salicylic acid.

**Conclusions**

Cryotherapy is more costly and no more effective than salicylic acid.

**Trial registration:**

Current Controlled Trials ISRCTN18994246 [controlled-trials.com] and National Research Register N0484189151.

## Background

Plantar warts (verrucae) are extremely common, and are experienced by most people at some time during their lives [1,2]. Many will spontaneously disappear without treatment [3]. However, treatment may be sought for a variety of reasons, such as discomfort or being prevented from undertaking sports or activities of daily living. There are a number of different treatments for plantar warts. In the UK the first line of treatment is generally an over-the-counter (OTC) salicylic acid preparation at strength of 15-26%. Second line treatments include cryotherapy treatment using liquid nitrogen and higher concentrations of salicylic acid for example 50% salicylic acid. Other treatments such as surgical curettage, complementary and alternative therapies are also available; however, there is a very little evidence to support the use of any of these treatments [4]. Even after treatment some plantar warts may fail to resolve, or may resolve and then reappear. Reported recurrence rates of cutaneous warts vary between 19% [5] following treatment with cryotherapy to around 30% [4] following surgical removal.

The treatment of warts represents a considerable cost burden to both patients and the NHS. In the literature [6], it has been indicated that, based on National Morbidity Survey data (1991-2), almost 2 million people in England and Wales see their General Practitioner (GP) for the treatment of cutaneous (non-genital) warts each year, at a cost of at least £40 million per annum. Economic analyses of salicylic acid and cryotherapy, alongside a wide range of other treatments in the UK setting, have been conducted by employing decision analytic modelling and synthesising data from a variety of sources [7,8]. These demonstrated that the most cost-effective treatments were over-the-counter (OTC) treatments; either salicylic acid or self-administered cryotherapy kits, bought by patients and applied in the patients' homes. Of the treatments provided in a primary care setting, the most cost-effective treatments were salicylic acid and cryotherapy delivered by a nurse. Both had similar cost-effectiveness values, but this was based on the assumption that patients would not receive more than three cryotherapy treatments, at two weekly intervals.

To date, no reports of a full economic evaluation of either salicylic acid or cryotherapy conducted based on the use of primary data, in a pragmatic setting, have been identified. This would increase the confidence in the conclusions on the cost-effectiveness of the treatments. Hence, an economic evaluation was conducted alongside a randomised controlled trial to investigate both the clinical and cost-effectiveness of two second line treatments; cryotherapy using liquid nitrogen compared with patient self-treatment with 50% salicylic acid. The clinical results of the trial have been published previously [9].

## Methods

### Study design and interventions

Full details of the study design and interventions have been described in the paper presenting the clinical results of the EVerT trial [9] and in the study protocol [10]. Briefly, a multicentre randomised controlled trial was conducted. Participants aged 12 years and over with a plantar wart were recruited from University podiatry school clinics, NHS podiatry clinics, and primary care in England, Scotland and Ireland. Participants were randomised to receive either up to a maximum of four cryotherapy treatments using liquid nitrogen, two to three weeks apart and delivered by a healthcare professional (podiatrist, practice nurse or General Practitioner), or patient daily self-treatment with 50% salicylic acid once they were directed on how to apply the treatment.

The primary outcome was complete clearance of all plantar warts at 12 weeks after randomization.

The study was approved by Trent multicentre Research Ethics Committee, Galway Research Ethics Committee and local ethics committees, Medicines and Healthcare Products Regulatory Agency, Irish Medicines Board and local Research and Development Trusts. All participants provided written informed consent prior to being enrolled in the study.

### Economic analysis

An economic evaluation was conducted at 12 weeks after randomisation of participants. The aim of the economic analysis was to assess the relative costs and effectiveness of cryotherapy and salicylic acid for the treatment of plantar warts. Trial data on both costs and effectiveness of the two comparators were synthesised to assess the additional cost required for an additional unit of outcome. For this analysis, a cost-effectiveness approach was taken, where the outcome was defined as complete clearance of plantar warts at 12 weeks. There were no utility data collected during the trial which would allow a cost-utility analysis to be undertaken. No discounting was applied given that the timeframe of the analysis was shorter than one year. The analysis was conducted on an "intention-to-treat" basis. Hence, the treatment groups were compared based on their original random allocation, regardless of protocol deviations and participants' compliance or withdrawal. The NHS perspective was taken for the analysis [11], where only costs directly linked to the NHS budget (e.g. GP or nurse visits, podiatrist time and cost of equipment and medications) were included.

### Outcome data

The outcome data used for the economic analysis was the complete clearance of plantar warts at 12 weeks. Clearance of plantar warts was defined as the restoration of normal skin upon close inspection. The data on the outcome were extracted primarily by two independent assessors, blinded to treatment allocation, from digital photographs of the participant's plantar warts taken at 12 weeks. An additional blinded outcome assessment at the recruiting site was undertaken at the participant's 12 week appointment. This assessment would be used in the case where the assessment of the digital photograph was not possible, for example, when the photograph was not interpretable or missing. If neither of these were available for a participant, then the patient's self-reported outcome recorded in the week 12 questionnaire was used.

### Resource usage data

During the participant's treatment period within the study, data on the resource usage component of the economic analysis were collected from both patients' self-completed questionnaires and the relevant questionnaires completed by the healthcare professionals. Participants were asked to complete the questionnaire about the number of visits to the clinic for treatment of their verruca and any other health service usage (for example, if they had seen a GP, practice nurse or attended an emergency visit with a GP due to their verruca). The number of visits to the podiatrist, nurse or GP for receiving the relevant treatment, as per the trial protocol and the randomisation arm, was recorded by the healthcare professional who treated the trial participant. In particular, details on the number of cryotherapy sessions administered and the number of tubes of salicylic acid provided to the patients were collected.

### Cost of the cryotherapy treatment

The cost of cryotherapy treatment comprised of three components: the cost of the equipment (i.e. the initial investment for purchasing the equipment), the cost of liquid nitrogen and the opportunity cost of the healthcare professional's time for attending the patients. The list of equipment required for cryotherapy is given in Table 1.

**Table 1:** Unit cost of cryotherapy equipment

Item	Source	Size/type	Price^a, b^	Average price	Price in £^c^	Price inc. VAT^d^
Cryogenic gloves*	BOC catalogue sent to a trial centre		€ 35	€ 35.00	£28.88	**£33.94**

Safety glasses*	BOC Products [12]		£4.42	£4.42	£4.42	**£5.19**

Dewar*	BOC catalogue sent to a trial centre	25 litre Aluminium	€ 833	€ 868.00	£716.30	**£841.65**
		25 litre Stainless Steel	€ 903			

Tipping trolley for dewar*	BOC catalogue sent to a trial centre		€ 433	€ 433.00	£357.33	**£419.86**

Withdrawal device*	BOC catalogue sent to a trial centre		€ 708	€ 708.00	£584.26	**£686.51**

Cryosurgery applicator*	BOC catalogue sent to a trial centre	330 ml capacity applicator	€ 630.40	€ 642.10	£529.88	**£622.61**
		450 ml capacity applicator	€ 653.80			

Slim probe*	BOC catalogue sent to a trial centre	1 mm	€ 99.40	€ 99.40	£82.03	**£96.38**
		2 mm	€ 99.40			
		3 mm	€ 99.40			

Cryogenic* apron	BOC Products [12]	Small	£137.28	£163.02	£163.02	**£191.55**
		Medium	£154.44			
		Large	£171.60			
		Extra large	£188.76			

^a ^Different prices are provided for different sizes/types of equipment. The average price was calculated

^b ^All the prices reported in Euro refer to 2009/2010 prices

^c ^Exchange rate: 1 € = 0.825232749 GBP (source: Google.co.uk, date 10/06/2010)

^d ^VAT was applied at 17.5%

*List compiled by a combination of interviewing podiatrists running the clinic and equipment bought to set up trial sites

Annuitization of the equipment cost was performed (Eq.1) for the economic analysis. The cost of the equipment (K), which was incurred upon their purchase, is spread over the lifetime of the equipment to obtain an equivalent annual cost (E). An interest rate (r) of 3.5% and a lifespan (n) for the cryogenic equipment of five years were used in the calculations of the annuity factor.

$$K=E\frac{1-{\left(1+r\right)}^{-n}}{r}$$

To assign an equipment cost per treatment session, the annual cost (E) was divided by the maximum number of treatments that can be provided by the healthcare professional. The maximum number of treatments was calculated based on an average appointment time of 20 min and, assuming full capacity of the clinics, for the total number of working days per year (i.e. 253 excluding bank holidays in the UK). The average appointment time of 20 min was based on the experience of podiatrists and practice nurses.

In addition to the equipment cost, the cost of liquid nitrogen, the freezing agent for the cryotherapy, was calculated. Liquid nitrogen is a liquid gas with high level of static evaporation at around 2% per day [13]. Hence, the liquid nitrogen dewars are refilled frequently, approximately every four to six weeks, even though the liquid nitrogen is not being used fully for patient treatments. It is, therefore, difficult to assess the quantity of liquid nitrogen that is required for a single treatment. However, from the trial data, it was noticed that in one trial centre which exclusively treated trial participants, four refills of a 25 litre dewar were ordered in a timeframe of three months. The cost of liquid nitrogen per treatment was calculated by dividing the cost of four refills of a 25 litre dewar by the total number of treatments performed in that centre. Liquid nitrogen costs are given in Table 2.

**Table 2:** Cost of liquid nitrogen

Item	Source	Price^a^		Average	Price in £^b^	Price inc. VAT^c^
Liquid nitrogen (calculated for 25 litre dewar)	Invoice to one of the trial centres	Invoice 1	€ 2.79/l			
		Invoice 2	€ 2.79/l			
		Invoice 3	€ 2.48/l			
		Invoice 4	€ 2.48/l	€ 65.88		
		
Delivery charges	Invoice to one of the trial centres	Invoice 1	€ 38/delivery			
		Invoice 2	€ 38/delivery			
		Invoice 3	€ 16.86/delivery			
		Invoice 4	€ 16.86/delivery	€ 27.43		

Total for liquid nitrogen and delivery				€ 93.31	£77.00	**£90.47**

^a ^All the prices reported in Euro refer to 2009/2010 prices

^b ^Exchange rate: 1 € = 0.825232749 GBP (source: Google.co.uk, date 10/06/2010)

^c ^VAT was applied at 17.5%

The clinician's time was calculated based on an average appointment time of 20 min. The treatments were administered to the trial participants either by a GP, a practice nurse or a podiatrist.

### Cost of the salicylic acid treatment

The cost of the salicylic acid treatment comprised of two components: the cost of the medication and the health professional's time spent for each treatment assessment visit. Unit costs for the salicylic acid medication, pads and plasters and calculation of the salicylic acid treatment costs are given in Table 3.

**Table 3:** Unit costs and calculation of the medication costs for the salicylic acid treatment

Item	Source	Price inc.VAT^a^
Verrugon 6 g^b^	BNF 59 [14]	**£3.00**

Fabric plasters^c^	Boots the chemist [15]^d^	**£1.49**

Pads^c^	Felt-pads. Product PPD126 [16]^d^	**£2.30**

^a ^VAT was applied at 17.5%^b ^Cost of Verrugon tubes was based on the number of entire tubes used^c ^The number of plasters and pads used was based on the number of applications applied, which in turn was based on the number of Verrugon tubes used. Patients using one tube of Verrugon required sufficient plasters and pads for 28 applications. The number of boxes of felt pads used per patient was calculated by dividing the number of applications by the total number of pads in a box (36), rounded up to a whole number. The number of plasters used was calculated by dividing the number of applications by the number of plasters in a box (20), rounded up to a whole number.^d ^Access date 10/06/2010

The cost of the healthcare professional's time for explaining treatment administration was calculated based on an average appointment time of 20 min.

### Unit costs of the treatments' components

The unit costs for the cryotherapy equipment were retrieved from the British Oxygen Company's (BOC) website [12] and from the BOC catalogue that was sent to the different trial centres. When more than one type of the same item was available, the average unit cost was calculated. The unit costs for the cryotherapy equipment are presented in Table 1.

The cost data that were used for calculating the cost of liquid nitrogen per treatment were retrieved from the purchases of liquid nitrogen at a single trial centre. The costs included the cost of the liquid nitrogen and the cost of delivery. The average cost over four purchases was calculated. Details are provided in Table 2.

For the salicylic acid treatment, the unit costs for the medication, pads and plasters are presented in Table 3.

### Unit costs of the healthcare professionals' time

The unit costs for the healthcare professional's time were retrieved from the Unit Costs of Health and Social Care (2009) document published by the Personal Social Services Research Unit (PSSRU) of Kent University [17]. The Unit Cost of healthcare professional's time was based on their speciality. Unit costs for healthcare professionals with the lowest qualifications were chosen. These are presented in Table 4.

**Table 4:** Unit costs for healthcare professionals' time

Healthcare professional	Source	Unit of measurement	Unit cost	Used for
Nurse (GP practice)	PSSRU [17]	Per hour (minute) in clinic	£28.00 (0.47)	Administration of cryotherapy/salicylic acid

Nurse (GP practice)	PSSRU [17]	Per surgery consultation	£10	Additional nurse visits

GP	PSSRU [17]	Per surgery/clinic minute	£2.70	Administration of cryotherapy/salicylic acid

GP	PSSRU [17]	Per surgery consultation lasting 11.7 min	£31	Additional GP visits

Community chiropodist/podiatrist	PSSRU [17]	Per clinic visit	£11.00	Administration of cryotherapy/salicylic acid

### Data analysis and presentation

The method of data analysis was mainly dictated by the level of missing data for the primary outcome. The base case analysis (scenario 1) was conducted as a "complete case analysis" where only patients with available primary outcome data were included. Where resource use data were missing, mean values were imputed (i.e. treatments visits, additional visits to the GP or nurse, number of cryotherapy treatments, number of tubes of Verrugon). For the visits to the treating healthcare professional, the mean imputation was performed based on the outcome group (i.e. verrucae gone or not gone) and the treatment allocation. For the cryotherapy treatments and the number of Verrugon tubes used, the means were imputed based on the outcome group only.

For this scenario, the analysis was conducted based on

1) unadjusted costs and outcomes

2) costs and outcomes adjusted for a number of covariates: age of the participants, whether or not they had received previous treatments and the type of plantar warts.

An additional analysis (scenario 2) was conducted by including all the patients (with and without primary outcome data). Data were imputed by using multiple imputation methods for the 11 patients who had missing primary outcome data. The multiple imputations were performed by using age, previous treatment and type of verrucae as covariates on both primary outcome and missing total costs.

For both analyses, the mean differences in costs and effects and the 95% confidence intervals around those were calculated by using bootstrap methods [18] (bias corrected and accelerated). For the mean difference in costs, a linear regression was used whilst for the difference in primary outcome logistic regression was used, given the binary nature of the data.

The cost-effectiveness of cryotherapy versus salicylic acid was assessed by comparing the incremental costs between the two arms of the trial to the incremental benefit which is expressed as the difference in the proportion of patients with completely cleared plantar warts at 12 weeks. The incremental cost-effectiveness ratio (ICER) was calculated by dividing the mean incremental cost (ΔC) by the mean incremental effect (ΔE) (ICER = ΔC/ΔE). For ease of interpretation of the results and to accommodate the uncertainty surrounding the estimation of costs and outcomes, the net monetary benefits were estimated for a range of willingness-to-pay thresholds (i.e. the amount that the decision maker is willing to pay for an additional unit of outcome) together with the uncertainty around the net benefit estimates. These are presented graphically using cost-effectiveness acceptability curves (CAECs) [19], which show the probability that the new intervention is cost-effective versus the comparator for a range of decision makers' willingness-to-pay thresholds for an additional unit of outcome.

All the analyses were conducted using STATA statistic and data analysis software, Version 10.1.

### Sensitivity analysis

Sensitivity analysis in the base case scenario was carried out by adopting an extreme approach whereby the administration of the treatment was assumed to be done by a nurse (rather than a GP) in those study sites that were set up in GP practices, and by excluding completely the cost of cryotherapy equipment and liquid nitrogen. The cost of salicylic acid was retained. In effect, this analysis would result in comparing both treatments based on the treatment visits and the cost of salicylic acid, rather than including the cost of cryotherapy medication as well. The decision to conduct this sensitivity analysis was led by the assumption that the costs will be driven by the healthcare professional's time and the cost of the treatment itself (i.e. the cost of the equipment and that of liquid nitrogen for the cryotherapy arm).

## Results

### Missing data

Missing data on resource usage relating to additional GP or nurse visits was 30% and 28% for the cryotherapy and salicylic acid groups, respectively. The level of missing data for treatment visits, number of tubes of salicylic acid and cryotherapy applications was much lower ranging from 2% to 7%.

The missing items were a result of either the trial participants not returning the questionnaire or not completing the relevant questions on the questionnaire. Missing data on number of treatment visits was due to missing podiatrist treatment assessment forms where these visits were recorded. The level of missing data was not related to the treatment allocation as demonstrated by a chi-squared test (Table 5).

**Table 5:** Missing data on resource use items and outcomes

	Missing response, n (%)		Treatment arm impact on level of missing data (Pearson chi-squared, p)
**Resource use item**	**Cryotherapy (n = 117)**	**Salicylic acid (n = 123)**	

Additional visits to GP or nurse	35 (30)	34 (28)	(0.1511, 0.697)

Treatment visits	8 (7)	3 (2)	(2.6528, 0.103)

Number of tubes of Verrugon	NA	7 (6)	NA

Number of cryotherapy treatments	8 (7)	NA	NA

**Outcome data**			

Primary outcome	7 (6)	4 (3)	(0.1345, 0.714)

NA Not applicable

### Resource use

Table 6 details the mean levels of resource usage per treatment arm. Participants in the cryotherapy arm had a mean of 3.59 visits to the GP, nurse or podiatrist for treatment. The salicylic acid arm participants had a mean of 1.94 visits. Only a small number of patients (three for both groups) had extra visits to the GP, in addition to the planned treatment visits. Participants in the cryotherapy arm had a mean of 0.04 additional visits to the GP, whilst those in the salicylic acid arm had a mean of 0.01 additional visits. Eight patients from both groups had additional visits to a nurse. This resulted in a mean number of additional nurse visits of 0.05 for the cryotherapy patients and 0.08 for the salicylic acid group. Salicylic acid patients received a mean of 1.25 tubes of salicylic acid, whilst cryotherapy patients received a mean of 3.49 treatments.

**Table 6:** Average resource usage

		Cryotherapy	Salicylic acid	Difference
**Average number of treatment visits**	N	109	120	
	
	Mean (SE)	3.59 (0.072)	1.94 (0.38)	1.65(p < 0.000)
	
	SD	0.75	0.42	
	
	Median (min, max)	4 (1, 5)	2 (1, 4)	
	
	Missing (%)	8 (7%)	3 (2%)	

**Average number of additional GP visits**	N	82	89	
	
	Mean (SE)	0.04 (0.03)	0.01 (0.01)	0.03(*p *= 0.376)
	
	SD	0.25	0.11	
	
	Median (min, max)	0 (0, 2)	0 (0, 1)	
	
	Missing (%)	35 (30%)	34 (28%)	

**Average number of additional nurse visits**	N	82	89	
	
	Mean (SE)	0.05 (0.03)	0.08 (0.04)	0.03(*p *= 0.530)
	
	SD	0.27	0.34	
	
	Median (min, max)	0 (0, 2)	0 (0, 2)	
	
	Missing (%)	35 (30%)	34 (28%)	

**Average number of tubes of Verrugon**	N	NA	116	NA
	
	Mean (SE)	NA	1.25 (0.04)	
	
	SD	NA	0.44	
	
	Median (min, max)	NA	1 (1, 2)	
	
	Missing (%)	NA	7 (6%)	

**Average number of cryotherapy treatments given to patients**	N	109	NA	NA
	
	Mean (SE)	3.49 (0.08)	NA	
	
	SD	0.80	NA	
	
	Median (min, max)	4 (1, 5)	NA	
	
	Missing (%)	8 (7%)	NA	

NA Not applicable

### Total costs

Table 7 summarizes the mean costs by item of resource usage, per treatment arm based on the base case analysis. The mean cost per patient in the cryotherapy and salicylic acid arm was approximately £150 and £49 respectively. The main driver of costs was the healthcare professionals' time for treatment administration, with the average cost per patient being larger in the cryotherapy group compared to the salicylic acid group (£88.69 vs. £39.59). The second largest cost for the cryotherapy group was the cost of treatment itself, which included the cost of equipment and liquid nitrogen. The average cost of the cryotherapy treatment per patient was £60.05.

**Table 7:** Costs by item of resource usage

Item	Treatment group	Mean cost (£)	SD (£)	Min-Max(£)
Treatment visits (Healthcare professional's time)	Salicylic acid	39.59	35.03	11.00-62.00
	Cryotherapy	88.69	74.46	11.00-270.00

Verrugon (inc.pads and plasters)	Salicylic acid	8.5	2.91	6.79-13.58

Cryotherapy cost (liquid nitrogen & equipment cost)	Cryotherapy	60.05	13.17	17.08-85.40

Additional visit to GP	Salicylic acid	0.35	2.84	0.00-31.00
	Cryotherapy	1.15	6.57	0.00-62.00

Additional visit to nurse	Salicylic acid	0.78	2.98	0.00-20.00
	Cryotherapy	0.49	2.31	0.00-20.00

**Total costs**	Salicylic acid	**49.22**	37.78	17.79-209.79
	Cryotherapy	**150.39**	78.45	50.08-365.40

**Difference = 101.17**	*p *< 0.001			

### Health outcomes

Details on the results of health outcomes analysis have been published in the clinical paper of this study [9]. Briefly, 17 of the 119 (14.3%) patients in the salicylic acid group and 15 of the 110 (13.6%) patients in the cryotherapy group had complete clearance of plantar warts at 12 weeks. There was no evidence of a difference between the salicylic acid and the cryotherapy groups (difference 0.65, 95% CI: -8.33-9.63, *p *= 0.89).

### Cost-effectiveness

The base case analysis (unadjusted and adjusted results), demonstrated that there is a significant difference in total costs between the two treatments. Cryotherapy costs on average £101.17 more per patient when compared with salicylic acid (Bias corrected and accelerated (BCA) 95% CI: 85.09-117.26) when using unadjusted estimates. The cost increased very slightly to £101.21, (BCA 95% CI 84.18-118.25), when adjusted estimates were used The treatment effect for cryotherapy is smaller than that of salicylic acid, although not statistically significant (difference of -0.0065, BCA 95%CI: -0.10-0.08 for the unadjusted analysis and -0.00336, BCA 95% CI: -0.09-0.08 for the adjusted analysis). In both cases, cryotherapy is a dominated alternative i.e. it is more costly and results in a smaller treatment effect compared with salicylic acid.

**Figure 1:**
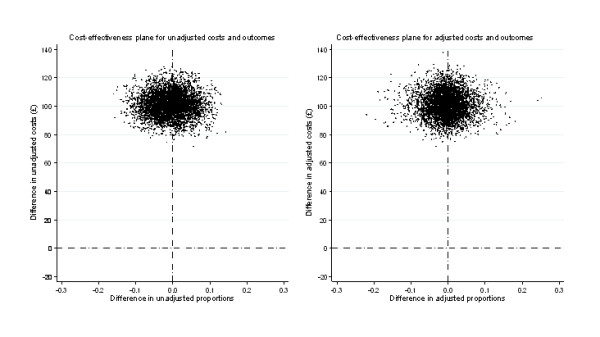
**Cost-effectiveness plane**.

**Figure 2:**
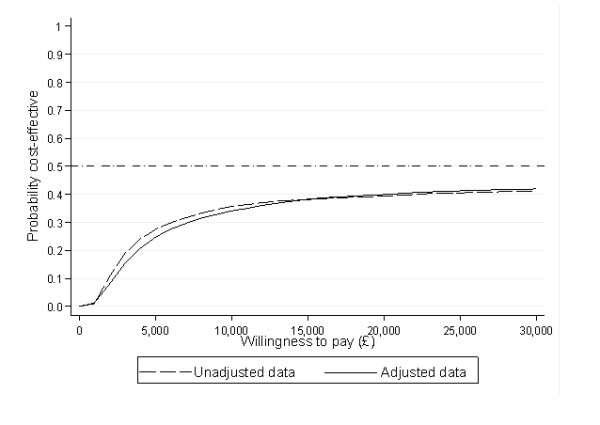
**Cost-effectiveness acceptability curve**.

Figure 1 shows the incremental costs (unadjusted & adjusted) and incremental effects for cryotherapy compared with salicylic acid on a cost-effectiveness plane. While there is little uncertainty around the cost estimates, this is not the case for the treatment effect estimates, where 50% of the simulations fall either side of the 0 point of *x-axis*. Figure 2 presents the cost-effectiveness acceptability curve (CEAC). This demonstrates the probability of cryotherapy being cost-effective given a specific willingness-to-pay value per cured patient. Both adjusted and unadjusted data give similar results: the probability of cryotherapy being cost-effective in relation to salicylic acid does not exceed 50% at a range of threshold willingness-to-pay values.

The scenario 2 analysis resulted in similar outcomes as the first analysis. Cryotherapy is more costly (incremental costs of £101.39, BCA 95% CI: 86.29-117.29) and less effective (incremental effects of -0.0083, BCA 95% CI: -0.10-0.08), hence it is a dominated alternative.

### Sensitivity analysis

The sensitivity analysis resulted in cryotherapy being £9.18, BCA 95% CI 7.09-11.26 (£9.17, BCA 95% CI 7.00-11.33) more costly than the salicylic group and having an incremental treatment effect of -0.0065, BCA 95% CI-0.10-0.08 (-0.0034 BCA 95% CI -0.09-0.08), based on unadjusted (adjusted) estimates. Cryotherapy is again more costly than the salicylic acid. This is due to the greater number of treatment visits that the patients made to the clinic, even though the time cost of the healthcare professional who administered the cryotherapy was lower (practice nurse vs. GP). This, in conjunction with the lack of treatment effect, leads to cryotherapy being a dominated option.

## Discussion

### Principal findings

The EVerT trial [9] has demonstrated that there is no evidence of a difference in effectiveness between cryotherapy and salicylic acid at 12 weeks. In fact, cryotherapy appears to be marginally worse than salicylic acid, however, these results did not reach statistical significance. Cryotherapy is also more expensive than salicylic acid, at an average incremental cost of approximately £101 per patient. This evidence results in cryotherapy being dominated (i.e. more costly, less effective) by salicylic acid. Both methods that were adopted for the data analysis (complete case and multiple imputation) resulted in the same conclusion as above. When excluding the cost of cryotherapy equipment and liquid nitrogen, and replacing the provision of the treatment from GP with practice nurse, as part of a sensitivity analysis, cryotherapy remained more expensive than the salicylic acid.

### Strengths and weaknesses

This analysis constitutes the first economic evaluation of treatments for plantar warts conducted based on primary data. It was conducted alongside a randomised controlled trial of pragmatic nature; hence the results should have strong external validity for the everyday clinical practice for plantar warts treatment in the UK setting.

The analysis took a narrow NHS perspective. A societal perspective would have provided a broader picture of the economic impact of the two treatments for plantar warts. However, given the lack of difference in effectiveness between the two treatments, incorporating additional costs such as those incurred by patients for travelling to the clinical practice to receive cryotherapy treatment would have only emphasised the conclusions of this analysis i.e. cryotherapy is a more costly option which does not offer additional benefits compared with salicylic acid.

There were missing data on both resource usage and the primary outcome. Whilst there were high missing data for self-reported health care use there were much fewer missing data on the main outcomes and main costs were much lower (below 10%). We do not think, therefore, this would have introduced significant bias to our results. The conclusions of the study could be compromised by the lack of these data; however, the analysis based on the complete cases on primary outcome and the one based on the multiple imputation, led to similar conclusions. This increases the confidence that the results are robust and not affected by the presence of missing data.

The economic analysis was conducted at 12 weeks after randomisation. The short timeframe might constitute a study limitation given the recurring nature of plantar warts. However, given the cure rates were similar between the two groups at six months it is likely that that the clinical results gathered over this short time frame are representative of a medium term follow-up. It would be of value to examine whether the conclusions of this analysis would still hold at a later time-point, such as one or two years after patients received the treatment and whether or not participants used health care resources at different rates. The only assumption that could possibly change the conclusions of this analysis is that patients treated with cryotherapy have a lower recurrence rate, hence consuming fewer additional resources for the treatment of the recurring warts.

We included an Irish site with 13 patients. Whilst in theory Irish patients may have had a different care pathway to UK patients in this instance it is unlikely as the Irish PI (CM) was an English podiatrist who had previously treated patients in Huddersfield so we do not anticipate that using the patients in the economic analysis would have contributed any bias to the results.

### Main drivers of the results

By excluding the cryotherapy treatment costs completely and reducing the cost of the healthcare professional who administers the treatment, it is made evident that the results are strongly driven by the lack of effectiveness as well as the larger number of treatment visits to the clinic that cryotherapy patients have. When the costs of the cryotherapy equipment and liquid nitrogen are included, it is expected to have even more negative results for the cost-effectiveness of cryotherapy. The combination of lack of difference in effectiveness between cryotherapy and salicylic acid and higher costs leads to cryotherapy being dominated by salicylic acid. Outside of a clinic setting the costs of salicylic acid would be even lower as it is a self-administered treatment. Consequently, including the costs of visits to a health care professional as used in the trial will over estimate the costs of using salicylic acid in usual practice.

### Recommendations for further research

The spectrum of the treatment options for plantar warts is quite wide, with most of them being over-the-counter treatments which patients can self-administer. The benefit of this type of administration is that the opportunity cost of healthcare professionals' time is reduced or eliminated completely, which leads to economic advantage. Given the results of EVerT trial which showed no difference in effectiveness between salicylic acid and cryotherapy, and the lower costs of salicylic acid, it would be valuable to have data from randomised clinical trials on the treatment effects of other over-the-counter treatments compared with salicylic acid. Even if these treatments do not prove to be dominant compared with salicylic acid (i.e. less costly and more effective), from the economics perspective it would still be relevant to examine the question on how much more we are prepared to pay for a better cure rate than the salicylic acid.

## Conclusions

Self-treatment with salicylic acid is more cost-effective for the treatment of plantar warts than cryotherapy administered by a healthcare professional. Whilst cryotherapy costs on average £101 more per participant over the 12 week time-frame, while there is no additional benefit, in terms of proportion of patients healed compared with salicylic acid. This evidence can contribute to the everyday clinical decision making process on which treatment should be used for plantar warts. More importantly, if there is a shared decision making between the healthcare professional and the patients themselves on the treatment pathway, this can be based on the full information provided to the patient that cryotherapy is not any better than salicylic acid while it costs more to the NHS.

## Abbreviations

BCA: Bias corrected and accelerated; BNF: British National Formulary; BOC: British Oxygen Company; GP: General Practitioner; OTC: Over-the-counter; PSSRU: Personal Social Services Research Unit.

## Competing interests

All authors have completed the Unified Competing Interest form at http://www.icmje.org/coi_disclosure.pdf (available on request from the corresponding author) and declare: SC, KH, SJ, GT, KT, MC, FH, NM and DT all received proportions of their salaries from the NIHR HTA grant in order to conduct the study. All other authors declare no support from any organisation for the submitted work. The 50% salicylic acid (Verrugon) plasters and felt pads were provided to the University of York, free of charge, by the manufacturer William Ransom & Son Plc. British Oxygen Corporation (BOC) Cryospeed provided liquid nitrogen storage equipment at reduced cost. Neither company has had any input into the design, analysis and reporting of the study.

## Authors' contributions

DT and JH wrote the original protocol. SC, MC, FH, NM, DT and KT were co applicants on the HTA application and refined the protocol which included the addition of the economic evaluation. ES designed and undertook the economic evaluation and acts as guarantor for the paper. DT and IW were the chief investigators and oversaw the study. CM, MC, NM, and FH recruited patients to the study and collected the trial data. SC and KH were the trial coordinators and GT the trial support officer who managed the trial on a day to day basis, assisted, SJ, ARK and CH with data validation and data cleaning. SJ, ARK and CH undertook the statistical analysis evaluating the clinical effectiveness of the interventions including the primary outcome used in the cost-effectiveness analysis. The initial draft of the manuscript was written by ES and SC, but KH, FH, DT, IW, KT, CM, GT, have been involved in revising it critically for important intellectual content. All authors read and approved the final manuscript.
